# On the Endermic Use of Purgatives

**Published:** 1845-12

**Authors:** 


### PRACTICAL MEDICINE, &c.

*On the Endermic Use of Purgatives.*—M. Salques makes the following practical remarks respecting the employment of purgatives:—

"There are many cases both of acute and chronic diseases, in which, although it is distinctly of importance to relieve the bowels, yet the irritability of the stomach is so great as to preclude the ordinary mode of exhibiting aperient medicine. Injections too frequently fail in their object, as they cannot pass the accumulation of hardened fecal matter. In such instances we have frequently recommended the recurrence to the endermic method." The following case exhibits the good effects of the plan:—A little girl, æt. 7, was attacked by acute meningitis. Constipation had existed for fifteen days, and the stomach rejected all medicine. Enemata could not be exhibited on account of the extreme aversion which the child showed to their administration. Two blisters had been applied to the thighs when M. Salques was called in, which he ordered to be sprinkled with powdered colocynth. Three hours afterwards the bowels were copiously relieved, and the cerebral symptoms vanished."

"M. M., æt. 76, was the subject of an apoplectic seizure. Seventeen days elapsed without relief, and the stomach rejected all medicine. Colocynth was, therefore, sprinkled on a blister behind the neck, and with the speedy effect of overcoming the constipation."

"M. D., æt. 82, had a slight apoplectic attack in January, 1844. Constipation became habitual, and after an accident caused by a fall, resisted calomel and even croton oil.—Hiccup and distention of the abdomen had supervened, when a blistered surface was sprinkled with colocynth. In five hours a prodigious quantity of fæces was passed."

From the analysis of these and similar cases the author draws the following deductions:—

1. There is a considerable number of cases in which the endermic method of purgation will be found highly advantageous.

2. Colocynth is peculiarly suited to the endermic application; the same good results do not follow the use of aloes.

3. If the application does not cause the action of the bowels, it should not be persisted in, as it is liable to irritate and inflame the bowels as much as if taken in the usual way.—*Revue Médicale de Dijon*, 1844, in *Ranking's Half-Yearly Abstract.*

